# Case Report: Adult-onset Still’s disease presenting with massive hemorrhagic pericardial effusion without the characteristic rash

**DOI:** 10.3389/fmed.2026.1848108

**Published:** 2026-06-04

**Authors:** Yun Li, Ning Wang, Junqi Hu, Changsheng Xie, Junchao Yang, Tingzhen Xu

**Affiliations:** 1 Department of Respiratory and Critical Care Medicine, The First Affiliated Hospital of Zhejiang Chinese Medical University (Zhejiang Provincial Hospital of Chinese Medicine), Hangzhou, Zhejiang, China; 2 The First School of Clinical Medicine, Zhejiang Chinese Medical University, Hangzhou, China

**Keywords:** adult-onset Still’s disease, case report, diagnostic challenge, diagnostic glucocorticoid therapy, hemorrhagic pericardial effusion

## Abstract

Adult-onset Still’s disease (AOSD) is a rare systemic autoinflammatory disorder without universally accepted disease-specific biomarkers, although serum ferritin and glycosylated ferritin may provide supportive diagnostic value. Therefore, diagnosis relies on a combination of clinical manifestations, laboratory abnormalities, and exclusion of alternative diagnoses, commonly using criteria such as Yamaguchi or Fautrel. Although serosal involvement has been reported, presentation with massive hemorrhagic pericardial effusion in the absence of the characteristic rash is extremely rare and may mimic tuberculosis, malignancy, or other rheumatic diseases. We report a 67-year-old Asian man who was referred to our department from a local hospital because of persistent cough, fever, and polyserositis predominantly characterized by massive hemorrhagic pericardial effusion. Laboratory evaluation revealed marked systemic inflammation, elevated ferritin levels, a positive interferon-gamma release assay, elevated adenosine deaminase in pericardial fluid, increased tumor markers, and anti-SAE1 positivity, initially raising suspicion for tuberculous pericarditis, malignancy, or autoimmune myopathy. However, comprehensive microbiological, cytological, imaging, and neuromuscular assessments found no evidence supporting these conditions, and the patient did not develop the typical evanescent rash of AOSD. After initiation of diagnostic glucocorticoid therapy, rapid defervescence and marked resolution of serous effusions were observed, supporting the diagnosis. This case highlights an atypical presentation of AOSD and emphasizes that AOSD should be considered in patients with unexplained serositis, particularly hemorrhagic pericardial effusion accompanied by systemic inflammation.

## Introduction

Adult-onset Still’s disease (AOSD) is a rare multisystem inflammatory disorder with an unclear pathogenesis, thought to involve aberrant activation of the innate immune system and excessive release of proinflammatory cytokines such as interleukin (IL)-1, IL-6, and IL-18 (1). The estimated incidence ranges from 0.16 to 0.4 per 100,000 individuals, making it an uncommon condition in routine clinical practice (2, 3). Its hallmark clinical features include recurrent fever, an evanescent rash, and arthritis or arthralgia, while other manifestations may involve hepatosplenomegaly, lymphadenopathy, pleuritis, and pericarditis (4). The Yamaguchi and Fautrel criteria are widely used for diagnosis and show high sensitivity and specificity (5, 6). However, no single universally accepted disease-specific biomarker is currently available for AOSD. Although elevated serum ferritin and reduced glycosylated ferritin may provide important supportive diagnostic value, diagnosis still relies largely on a combination of clinical manifestations, laboratory findings, and exclusion of infections, malignancies, and other rheumatic diseases (7). Such delay may lead to unnecessary anti-tuberculosis therapy, extensive oncologic evaluation, or postponed immunomodulatory treatment.

Fever and the characteristic rash are generally regarded as key diagnostic features of AOSD (8, 9), but the rash may be absent or atypical in some patients, thereby increasing diagnostic difficulty, particularly in resource-limited settings where this hallmark manifestation may facilitate early clinical recognition (10). Serosal involvement has been reported (11), yet presentation with polyserositis, particularly massive hemorrhagic pericardial effusion without rash, is extremely rare and may mimic tuberculous pericarditis or malignancy. we describe a case of AOSD presenting with this atypical manifestation and discuss its diagnostic challenges.

## Case description

### Clinical presentation

A 67-year-old Asian man with no significant past medical history and no known history of tuberculosis, malignancy, or rheumatic autoimmune disease, was admitted with a 6-day history of fever (38.8 °C) and a 2-month history of productive cough. He denied recent weight loss. Initial imaging at a local hospital suggested pulmonary infiltrates and a small pericardial effusion. Empirical antibiotic therapy was ineffective, and the patient was subsequently referred to our hospital.

On admission to our hospital, the patient had persistent fever (39.1 °C) without chest pain, dyspnea, or abdominal symptoms. Physical examination revealed pharyngeal congestion, tonsillar swelling, and coarse breath sounds bilaterally. Mild erythematous swelling of the proximal interphalangeal and metacarpophalangeal joints of both hands was noted, without tenderness, limitation of movement, or muscle weakness. No rash was observed.

### Laboratory findings and diagnosis

Laboratory investigations demonstrated leukocytosis (white blood cell count: 13.2*10⁹/L, normal range: 3.5–9.5 *10⁹/L) with marked neutrophilia (absolute neutrophil count: 12.6 *10⁹/L, normal range: 1.8–6.3*10⁹/L), mild anemia (hemoglobin: 116 g/L, normal range: 130–175 g/L), and thrombocytosis (platelet count: 435 *10⁹/L, normal range: 125–350 *10⁹/L). Inflammatory markers were markedly elevated, including C-reactive protein (CRP: 91.78 mg/L, normal value: <6 mg/L) and erythrocyte sedimentation rate (ESR: 60 mm/h, normal value: <15 mm/h). Serum ferritin was significantly increased (1725 ng/mL, normal range: 30–400 ng/mL). Liver function tests revealed elevated aspartate aminotransferase (AST: 100 U/L, normal value: <40 U/L) and alanine aminotransferase (ALT: 70 U/L, normal value: <50 U/L). Lactate dehydrogenase (LDH: 242 U/L, normal range: 120–250 U/L) and creatine kinase (CK: 55 U/L, normal range: 38–174 U/L) were within normal limits.

Tuberculosis screening revealed a positive interferon-gamma release assay and a weakly positive tuberculin skin test. Autoimmune testing demonstrated antinuclear antibody (ANA) positivity at a titer of 1:100, with anti-PM-Scl and anti-SAE1 antibodies. Rheumatoid factor was within the normal range, and other myositis-related antibodies were negative. Metagenomic sequencing detected low-level reads of *Enterococcus faecium* and Epstein–Barr virus.

Contrast-enhanced chest CT revealed bilateral pulmonary infiltrates, small pleural effusions, moderate-to-large pericardial effusion, and multiple calcified mediastinal and hilar lymph nodes (Figure 1). Despite empirical intravenous antibiotic therapy with levofloxacin (0.5 g once daily), followed by teicoplanin (0.4 g once daily), the patient remained febrile with progressive pleural and pericardial effusions, making bacterial infection less likely.

**Figure 1:**
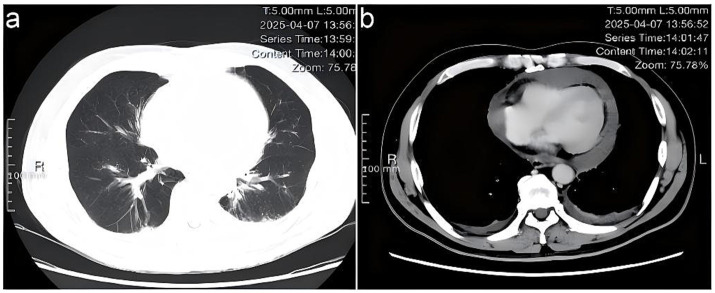
Imaging examinations. **(a)** CT: scattered inflammatory infiltrates in both lungs. **(b)** CT: mild bilateral pleural effusions and a moderate-to-large pericardial effusion.

Pericardiocentesis yielded hemorrhagic fluid (total 1,550 mL), which was exudative with lymphocyte predominance (77%) and elevated adenosine deaminase (ADA: 46 U/L, normal value: <40 U/L). Tumor markers in the fluid (CA125, CYFRA21-1, and ferritin) were markedly elevated; however, cytology and histopathology showed no evidence of malignancy. PET-CT further supported a non-malignant process, demonstrating diffuse inflammatory changes involving the pericardium, lungs, and lymph nodes (Figure 2). Taken together, these findings were most consistent with adult-onset Still’s disease.

**Figure 2:**
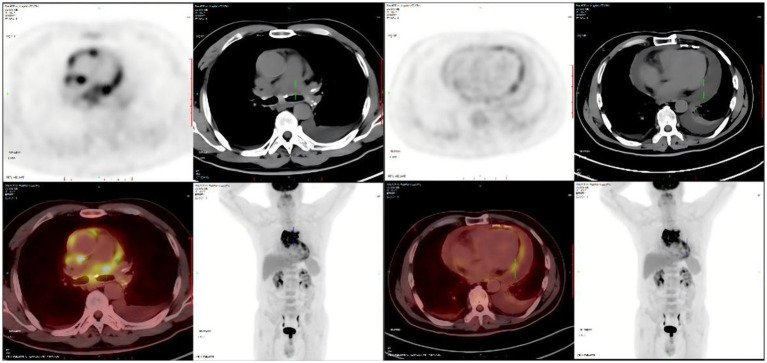
Imaging examinations: PET-CT: inflammatory changes affecting the pericardium, both lungs, mediastinal and hilar lymph nodes, and cervical lymph nodes.

### Diagnostic assessment

The diagnostic process was challenging due to overlapping clinical and laboratory features. Elevated adenosine deaminase levels in the pericardial fluid and a positive interferon-gamma release assay initially suggested tuberculous pericarditis, while hemorrhagic pericardial effusion with markedly increased tumor markers raised concern for an underlying malignancy. Lymph node biopsy was recommended to further assess the mediastinal and hilar lymphadenopathy; however, the patient declined the procedure. Nevertheless, repeated microbiological investigations, cytological examination of the pericardial fluid, and whole-body PET-CT showed no evidence strongly suggestive of lymphoma or other malignancy.

The diagnostic process was challenging because tuberculous pericarditis and malignancy were initially strongly suspected based on elevated ADA levels, positive IGRA, hemorrhagic effusion, and increased tumor markers. Lymph node biopsy was recommended but declined by the patient. However, repeated microbiological tests, cytology, and PET-CT did not confirm infection or malignancy. Considering the persistent systemic inflammatory syndrome and marked hyperferritinemia, AOSD was considered the most likely diagnosis.

### Treatment and follow-up

Given that tuberculosis could not be completely excluded because the patient declined pericardial biopsy and bronchoscopy, glucocorticoid therapy was initiated after comprehensive evaluation and informed consent. Administration of methylprednisolone (40 mg/day) was followed by a dramatic clinical response within 48 h, including resolution of fever, a marked reduction in pericardial drainage, and significant decreases in CRP and ferritin levels (Figure 3a). The patient was subsequently discharged on oral methylprednisolone (28 mg/day), which was gradually tapered according to clinical and laboratory response. By the follow-up in January 2026, the patient had achieved complete clinical and laboratory remission. Chest CT showed marked interval improvement, with resolving pulmonary infiltrates, cleared serous effusions, and stable mediastinal lymph nodes compared to April 8, thereby validating the initial diagnosis and therapeutic approach. It is worth noting that the previously reported suspicious pulmonary interstitial changes have also completely resolved (Figures 3b,c).

**Figure 3:**
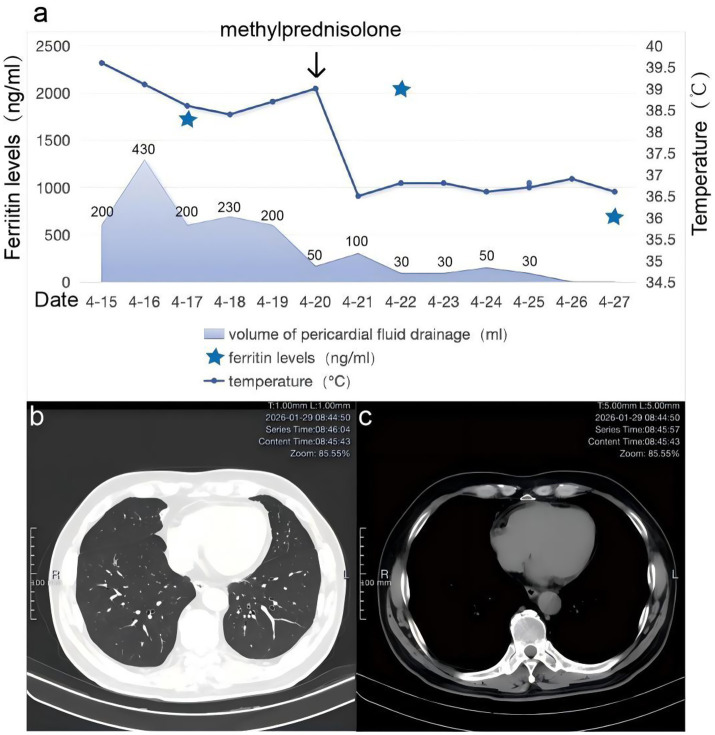
Therapeutic efficacy was demonstrated by clinical response. **(a)** Changes in body temperature, serum ferritin levels, and pericardial effusion drainage volume. **(b,c)** CT: Resolution of bilateral pulmonary infiltrates and serous effusions with stable mediastinal lymphadenopathy.

## Discussion and conclusion

Adult-onset Still’s disease (AOSD) remains a diagnostic challenge because no universally accepted disease-specific biomarker is available, although markedly elevated serum ferritin and reduced glycosylated ferritin may provide useful supportive diagnostic information (8). Diagnosis relies on a combination of clinical manifestations, laboratory abnormalities, classification criteria, and exclusion of alternative conditions.

The Yamaguchi criteria are the most commonly used diagnostic tool, with a reported sensitivity of approximately 93.5% (5, 12). In the present case, persistent fever, leukocytosis with neutrophilia, elevated liver enzymes, hyperferritinemia, and polyserositis supported the diagnosis. However, the absence of the characteristic evanescent rash, low-titer ANA positivity, and marked serosal involvement, particularly massive hemorrhagic pericardial effusion, initially redirected diagnostic consideration toward tuberculosis, malignancy, or other autoimmune diseases.

Because of a positive TB-IGRA result and elevated ADA levels, tuberculous pericarditis was strongly suspected. However, ADA is not specific for tuberculosis and may also be elevated in various inflammatory conditions (13). Repeated microbiological examinations failed to detect *Mycobacterium tuberculosis*, indicating that elevated ADA combined with a positive IGRA is insufficient to establish the diagnosis and may lead to unnecessary anti-tuberculosis treatment.

Malignancy represents another important differential diagnosis in patients with hemorrhagic pericardial effusion. In this case, markedly elevated CA125 and CYFRA21-1 levels in the pericardial fluid raised concern for an underlying malignancy. Nevertheless, neither cytological examination of the effusion nor whole-body PET-CT imaging revealed any evidence of tumor. Previous studies have shown that tumor markers in serous effusions lack specificity, as they may increase significantly in both acute and chronic inflammatory conditions (14). Therefore, these markers should not be used as the sole criteria to distinguish benign from malignant effusions.

Sarcoidosis was also considered because it may present with fever, cough, mediastinal or hilar lymphadenopathy, pulmonary infiltrates, and occasionally serosal involvement including pericardial effusion (15). Because sarcoidosis also responds to glucocorticoids, clinical improvement after steroid therapy does not exclude this disease. Diagnosis generally requires histopathological evidence of non-caseating granulomas (16). However, the patient lacked other typical clinical features, and whole-body PET-CT did not demonstrate findings strongly suggestive of active multisystem granulomatous disease. Although tissue confirmation was not obtained, the available clinical and imaging findings made sarcoidosis less likely.

Another diagnostically important feature of this case was the immunologic profile. The patient showed ANA positivity at a low titer (1:100), which is below the threshold usually considered clinically significant in many autoimmune connective tissue diseases (commonly ≥1:160). Previous studies have shown that low-titer or transient autoantibody positivity may occur in systemic inflammatory states and does not exclude AOSD (17). Anti-SAE1 antibodies are relatively specific for dermatomyositis and therefore raised concern for autoimmune myopathy, particularly clinically amyopathic dermatomyositis (CADM), given the chronic cough and pulmonary infiltrative abnormalities on chest imaging, although a definite fibrosing ILD pattern was not demonstrated (18). In addition, pulmonary involvement in AOSD itself is uncommon and generally rarer than serosal involvement, with reported prevalence of approximately 5–12%, but may be associated with worse outcomes (19). However, the patient had no heliotrope rash, Gottron’s papules or sign, proximal muscle weakness, dysphagia, or other characteristic cutaneous manifestations. Serum creatine kinase remained within the normal range (55 U/L). Electromyography showed no evidence of inflammatory myopathy and demonstrated only bilateral ulnar neuropathy. Moreover, the radiologic findings were not typical of the peripheral consolidative pattern reported in AOSD-associated lung disease. Follow-up thin-section chest CT performed in January 2026 demonstrated complete resolution of the previous pulmonary abnormalities without persistent interstitial changes. Taken together, these findings made dermatomyositis/CADM less likely and favored transient inflammatory pulmonary involvement rather than established connective tissue disease-related or AOSD-associated interstitial lung disease. Nevertheless, continued clinical follow-up remains warranted.

AOSD often presents with heterogeneous and overlapping features, and reliance on isolated laboratory findings may cause diagnostic bias; therefore, comprehensive clinical evaluation and exclusion of alternative diagnoses are essential.

Notably, this patient did not develop the characteristic salmon-colored rash, one of the typical triad features of AOSD. The reported incidence of skin rash in AOSD ranges from 51 to 87% (mean 72.7%), indicating that it is not a mandatory diagnostic criterion (20). The absence of this characteristic sign significantly increases diagnostic difficulty and requires greater reliance on exclusion strategies and supportive laboratory findings.

In addition to the absence of rash, the most striking feature of this case was massive hemorrhagic pericardial effusion, a relatively uncommon manifestation of AOSD. Serosal involvement in AOSD may present as pleural, pericardial, or peritoneal effusions. Approximately 10–25% of patients develop cardiac involvement, with pericarditis being the most frequent manifestation and, in severe cases, progressing to cardiac tamponade (9, 21). However, initial presentation with massive hemorrhagic pericardial effusion is extremely rare. Previous reports have described only a few cases of AOSD with polyserous effusions or isolated pericardial effusion. Sheikh et al. reported a case dominated by massive serositis, where both serum ferritin and pleural fluid ADA were markedly elevated. The patient responded well to high-dose glucocorticoids and an IL-1 receptor antagonist (22). Yoo et al. described a case where pericardial effusion appeared during disease relapse and was followed by fever, sore throat, arthritis, and rash; symptoms improved after glucocorticoid and aspirin therapy (23).

In clinical practice, massive hemorrhagic pericardial effusion often prompts consideration of malignancy or tuberculosis as more common causes, which may delay the correct diagnosis. Generally, tuberculous pericarditis presents with lymphocyte-predominant exudative effusion accompanied by elevated ADA levels (24), whereas malignant pericardial effusion may reveal tumor cells on cytological examination (25). Autoimmune diseases such as systemic lupus erythematosus may also present with serositis (26). Therefore, after excluding infectious, malignant, and other rheumatic diseases, AOSD should also be considered in patients with prominent systemic inflammation and markedly elevated serum ferritin levels.

Although rare, when massive hemorrhagic pericardial effusion is the initial finding, malignancy or tuberculosis is usually suspected, which can delay correct diagnosis. Bodard et al. analyzed 96 AOSD cases and found that cardiac involvement was associated with higher inflammatory markers and more frequent ICU admissions. It was identified as an independent predictor of poor prognosis (27). Therefore, early recognition and treatment are crucial to prevent life-threatening complications.

Glucocorticoids remain the first-line treatment for acute AOSD, and many patients respond rapidly. In the present case, methylprednisolone therapy was followed by prompt clinical and laboratory improvement. However, treatment response alone should not be considered diagnostic, as lymphoma (including angioimmunoblastic T-cell lymphoma), occult malignancy, or infectious diseases such as early tuberculous pericarditis may also transiently improve after glucocorticoid exposure. Therefore, close follow-up and continued reassessment are essential. After discharge, the patient was transitioned to oral methylprednisolone with gradual tapering, which was completely discontinued by January 2026. At follow-up, the patient remained clinically stable without recurrent fever, recurrent serosal effusion, or emergence of alternative diagnoses, and follow-up imaging showed sustained improvement.

For steroid-dependent or refractory disease, methotrexate may serve as a corticosteroid-sparing agent. In patients with inadequate response or intolerance to conventional therapy, biologic agents targeting IL-1 or IL-6 (28, 29), including anakinra, tocilizumab, and sarilumab, have shown clinical benefit, with sarilumab also reported to facilitate glucocorticoid tapering. In severe cases complicated by refractory macrophage activation syndrome, emerging IL-18-targeted therapies may offer an additional therapeutic option (30). Nevertheless, longer-term surveillance remains warranted in atypical presentations such as massive hemorrhagic pericardial effusion.

In summary, this case illustrates a rare presentation of AOSD characterized by massive hemorrhagic pericardial effusion without the typical rash, leading to significant diagnostic difficulty. AOSD should be considered in patients with unexplained hemorrhagic pericardial effusion after exclusion of infection and malignancy. Early recognition, careful differential diagnosis, and timely immunomodulatory therapy are essential for improving outcomes.

## Patient perspective

The patient reported rapid relief of symptoms following glucocorticoid therapy, particularly resolution of fever and chest discomfort, and expressed satisfaction with the treatment outcome.

## Data Availability

The original contributions presented in the study are included in the article/Supplementary material, further inquiries can be directed to the corresponding authors.
