# Supplementary material for: Case Report: Adult-onset Still’s disease presenting with massive hemorrhagic pericardial effusion without the characteristic rash
Source: Front Med (Lausanne). 2026 Jun 4;13:1848108. doi: 10.3389/fmed.2026.1848108 (PMC13275211; doi:10.3389/fmed.2026.1848108)
Supplement: Supplementary file 1 [file Data_Sheet_1.pdf]

CARE
case report guidelines

CARE Checklist of information to include when writing a case report

(cc) BY-NC-ND

Topic	Item	Checklist item description	Reported on Line
**Title**	1	The diagnosis or intervention of primary focus followed by the words "case report"	Title
**Key Words**	2	2 to 5 key words that identify diagnoses or interventions in this case report, including "case report"	Keywords
**Abstract (no references)**	3a	Introduction: What is unique about this case and what does it add to the scientific literature?	Abstract
	3b	Main symptoms and/or important clinical findings	Abstract
	3c	The main diagnoses, therapeutic interventions, and outcomes	Abstract
	3d	Conclusion—What is the main "take-away" lesson(s) from this case?	Abstract
**Introduction**	4	One or two paragraphs summarizing why this case is unique (**may include references**)	Introduction
**Patient Information**	5a	De-identified patient specific information.	Introduction
	5b	Primary concerns and symptoms of the patient.	Introduction
	5c	Medical, family, and psycho-social history including relevant genetic information	Introduction
	5d	Relevant past interventions with outcomes	Introduction
**Clinical Findings**	6	Describe significant physical examination (PE) and important clinical findings.	Clinical resentation
**Timeline**	7	Historical and current information from this episode of care organized as a timeline	Figure.3
**Diagnostic Assessment**	8a	Diagnostic testing (such as PE, laboratory testing, imaging, surveys).	Laboratory findings and diagnosis
	8b	Diagnostic challenges (such as access to testing, financial, or cultural)	Diagnostic assessment
	8c	Diagnosis (including other diagnoses considered)	Diagnostic assessment
	8d	Prognosis (such as staging in oncology) where applicable	Diagnostic assessment
**Therapeutic Intervention**	9a	Types of therapeutic intervention (such as pharmacologic, surgical, preventive, self-care)	Treatment and follow-up
	9b	Administration of therapeutic intervention (such as dosage, strength, duration)	Treatment and follow-up
	9c	Changes in therapeutic intervention (with rationale)	Treatment and follow-up
**Follow-up and Outcomes**	10a	Clinician and patient-assessed outcomes (if available)	Treatment and follow-up
	10b	Important follow-up diagnostic and other test results	Treatment and follow-up
	10c	Intervention adherence and tolerability (How was this assessed?)	Treatment and follow-up
	10d	Adverse and unanticipated events	Treatment and follow-up
**Discussion**	11a	A scientific discussion of the strengths AND limitations associated with this case report	Discussion and conclusion
	11b	Discussion of the relevant medical literature **with references**.	Discussion and conclusion
	11c	The scientific rationale for any conclusions (including assessment of possible causes)	Discussion and conclusion
	11d	The primary "take-away" lessons of this case report (without references) in a one paragraph conclusion	Discussion and conclusion
**Patient Perspective**	12	The patient should share their perspective in one to two paragraphs on the treatment(s) they received	Patient Perspective
**Informed Consent**	13	Did the patient give informed consent? Please provide if requested	**Yes** ☑ **No** ☐
